# Controlled human exposure to diesel exhaust: results illuminate health effects of traffic-related air pollution and inform future directions

**DOI:** 10.1186/s12989-022-00450-5

**Published:** 2022-02-09

**Authors:** Erin Long, Christopher Carlsten

**Affiliations:** 1 Faculty of Medicine, University of British Columbia, 317 – 2194 Health Sciences Mall, Vancouver, BC V6T 1Z3 Canada; 2 Division of Respiratory Medicine, Department of Medicine, University of British Columbia, 2775 Laurel Street 7th Floor, Vancouver, BC V5Z 1M9 Canada

**Keywords:** Controlled human exposure, Diesel exhaust, Particulate matter, Air pollution, Humans

## Abstract

Air pollution is an issue of increasing interest due to its globally relevant impacts on morbidity and mortality. Controlled human exposure (CHE) studies are often employed to investigate the impacts of pollution on human health, with diesel exhaust (DE) commonly used as a surrogate of traffic related air pollution (TRAP). This paper will review the results derived from 104 publications of CHE to DE (CHE-DE) with respect to health outcomes. CHE-DE studies have provided mechanistic evidence supporting TRAP’s detrimental effects on related to the cardiovascular system (e.g., vasomotor dysfunction, inhibition of fibrinolysis, and impaired cardiac function) and respiratory system (e.g., airway inflammation, increased airway responsiveness, and clinical symptoms of asthma). Oxidative stress is thought to be the primary mechanism of TRAP-induced effects and has been supported by several CHE-DE studies. A historical limitation of some air pollution research is consideration of TRAP (or its components) in isolation, limiting insight into the interactions between TRAP and other environmental factors often encountered in tandem. CHE-DE studies can help to shed light on complex conditions, and several have included co-exposure to common elements such as allergens, ozone, and activity level. The ability of filters to mitigate the adverse effects of DE, by limiting exposure to the particulate fraction of polluted aerosols, has also been examined. While various biomarkers of DE exposure have been evaluated in CHE-DE studies, a definitive such endpoint has yet to be identified. In spite of the above advantages, this paradigm for TRAP is constrained to acute exposures and can only be indirectly applied to chronic exposures, despite the critical real-world impact of living long-term with TRAP. Those with significant medical conditions are often excluded from CHE-DE studies and so results derived from healthy individuals may not apply to more susceptible populations whose further study is needed to avoid potentially misleading conclusions. In spite of limitations, the contributions of CHE-DE studies have greatly advanced current understanding of the health impacts associated with TRAP exposure, especially regarding mechanisms therein, with important implications for regulation and policy.

## Background

Air pollution, one of the leading causes of death worldwide, is linked to an estimated 7 million deaths per year [1]. Numerous adverse health outcomes are associated with air pollution affecting the cardiovascular, respiratory, and neurological systems [2, 3]. Air quality is a persistent and growing global issue as humans continue to generate pollution through industrial sources, vehicles, and household energy consumption. While agencies such as the World Health Organization (WHO) [4, 5] and the US Environmental Protection Agency [6] have set standards for air quality, in 2019 over 90% of the world’s population lived in regions with pollutant levels above 2005 WHO air quality standards [4]. Traffic related air pollution (TRAP) constitutes a substantial portion of air pollution globally, with a significant source of TRAP being diesel engines, used in vehicles such as trucks, buses, boats, cars, vans, and trains.

The health effects of diesel exhaust (DE) are commonly investigated in controlled human exposure (CHE) studies. In this experimental design, participants are exposed to a known quantity of pollutant under controlled conditions, typically inside a specialized exposure chamber. For CHE to DE (CHE-DE) studies, DE exposure is typically quantified by the concentration of particulate matter (PM) with diameter less than 2.5 μm (PM_2.5_) or, less frequently, 10 μm (PM_10_) as these are common PM metrics linked in voluminous epidemiological studies to date [7]. In addition to PM, DE is composed of gases, notably nitrogen dioxide (NO_2_), carbon monoxide (CO), and gaseous hydrocarbons [8] which are often measured but not titrated as the primary exposure metric in these studies. CHE-DE are valuable for studying the acute effects of DE exposure due to their ability to control exposure duration, PM concentration, and other exposure characteristics such as humidity and temperature. Furthermore, the use of individual participants as their own control, given the crossover design, is a powerful factor in effectively eliminating confounding factors that may vex other study designs. This crossover design also facilitates clear statistical analysis of such variables (sex, age, baseline health-related phenotypic characteristics, host genotype, etc.) as potential modifiers of the primary effect of DE across a range of endpoints. The design of CHE-DE experiments is conducive to analysis of wide variety of endpoints derived from blood, urine, or airway samples, or in terms of a range of physiologic parameters derived from respiratory or cardiovascular outputs, or from repeated administration of questionnaires.

These CHE-DE studies have investigated a broad range of health outcomes, from cardiovascular effects such as vasomotor function and thrombosis, to pulmonary effects including inflammation and lung function. Mechanisms of DE-associated health effects on the genetic, epigenetic, and biochemical levels have also been studied in CHE-DE papers. As the set-up of CHE-DE studies makes it possible to control concurrent exposures, many papers have investigated the influence of co-exposures such as ozone (O_3_) and allergens on the impact of DE inhalation. While findings from CHE-DE studies have been previously reviewed [9–11] the literature with respect to CHE-DE has grown profoundly in recent years and continues to do so. Thus, the focus of this paper is to provide a broad, updated review of the health-related results gleaned from this body of literature.

## Methods

A literature search was conducted using the PubMed and Web of Science databases. The keyword “diesel exhaust” or “was included in all search queries, in combination with “controlled human exposure”, “human exposure”, or “exposure” (e.g., “diesel exhaust” AND “human exposure”). Eligible studies must have exposed participants to a controlled quantity of DE via inhalation and been published in December 2020 or earlier. We excluded letters, abstracts, and academic theses. The reference lists of included articles as well as the Clinicaltrials.gov registration page for publications that reported a clinical trial number were also searched for eligible studies. Through this process, we identified 104 publications eligible for review. The methodology of each paper, such as the DE concentration, gaseous composition, exposure conditions, and exposure durations is reviewed in detail in a separate companion paper currently under review. Main findings with respect to health outcomes were extracted and summarized in a table together with brief details of study methodology (PM concentration(s) of DE exposures, concurrent exposures, and participant characteristics). Based on their main findings, eligible publications were then categorized by a primary topic: oxidative stress and antioxidants, systemic inflammation, respiratory, cardiovascular, neurological, exercise, co-exposures, filtered DE, markers and quantification of DE exposure, and other. Detailed evaluation of each paper was then conducted to synthesize this review.

## Results

**Fig. 1:**
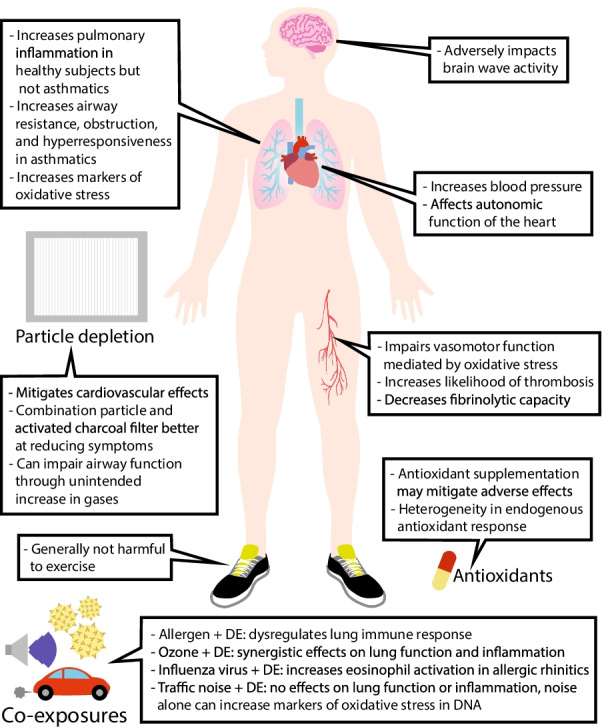
Summary of health outcome findings from controlled human exposure to diesel exhaust studies. *DE* diesel exhaust

Main health outcomes of the 104 reviewed publications along with an abbreviated description of study methodology are outlined in Table 1. 24 studies included co-exposure to DE and additional agents such as allergens (6 studies), antioxidants (5 studies), ozone (3 studies), and various other agents. 46 studies included healthy participants only, 19 studies included participants with asthma or atopy, 7 studies included participants with metabolic syndrome, and 7 studies included participants with other morbidities such as heart failure [94, 95], COPD [26, 35], and coronary artery disease [51, 59, 69]. Further details of study methodology can be found in a separate companion paper which is currently under review. With respect to the primary category of health outcomes reported, 9 publications reported oxidative stress outcomes, 3 publications reported systemic inflammation outcomes, 25 publications reported respiratory outcomes, 23 publications reported cardiovascular outcomes, 2 publications reported neurological outcomes, 8 publications reported exercise outcomes, 12 publications reported co-exposure outcomes, 7 publications reported filtered DE outcomes, and 15 publications reported outcomes that did not fall under any of the above categories. Fig. 1 provides a brief summary of all health outcomes in the reviewed articles.

**Table 1:** Main health outcome findings and brief details of methodology from reviewed publications

Study	Nominal PM concentration of DE exposures (μg/m^3^)	Concurrent exposures	Participant characteristics	Main findings	Primary topic
Cosselman et al. [12]	PM_2.5_ = 200	Antioxidant	Healthy	Acute DE exposure associated with oxidative changes in healthy volunteers DE significantly decreased GSH/GSSG ratio and significantly increased IL-6 mRNA Antioxidant pre-treatment did not significantly attenuate DE effect on GSH/GSSG ratio, and non-significantly decreased DE effect on IL-6 mRNA	Oxidative stress and antioxidants
Carlsten et al. [13]	PM_2.5_ = 300	Antioxidant	Healthy Asthmatics	Antioxidant supplementation decreased baseline airway hyperresponsiveness in hyperresponsive subjects DE exposure significantly increased airway hyperresponsiveness in hyperresponsive subjects DE-induced increase hyperresponsiveness was attenuated by antioxidant supplementation	Oxidative stress and antioxidants
Yamamoto et al. [14]	PM_2.5_ = 300	Antioxidant	Asthmatics	Acute DE exposure causes changes in systemic miRNAs DE associated changes in miR-144 may be mediated by oxidative stress	Oxidative stress and antioxidants
Pettit et al. [15]	PM_2.5_ = 300	None	Healthy	DE exposure was associated with changes in expression of genes linked to oxidative stress, protein degradation, and coagulation pathways	Oxidative stress and antioxidants
Allen et al. [16]	PM_2.5_ = 200	None	Metabolic syndrome	DE exposure did not induce changes in markers of oxidative stress or systemic antioxidant response in subjects with metabolic syndrome	Oxidative stress and antioxidants
Peretz et al. [17]	PM_2.5_ = 50, 100, 200 (multi-concentration crossover)	None	Healthy	DE exposure associated with changes in gene expression in peripheral blood mononuclear cells Genes associated with oxidative stress and inflammatory pathways are involved	Oxidative stress and antioxidants
Pourazar et al. [18]	PM_10_ = 300	None	Healthy	DE exposure activated transcription factors associated with oxidative stress, inducing increased production of proinflammatory cytokines	Oxidative stress and antioxidants
Mudway et al. [19]	PM_10_ = 100	None	Healthy	Airway inflammation nor antioxidant depletion was observed in airways 6 h post DE exposure Reduced glutathione was increased in bronchial and nasal airways at 6 h post-DE exposure DE demonstrated oxidative activity in vitro	Oxidative stress and antioxidants
Blomberg et al. [20]	PM = 300	None	Healthy	DE exposure increased ascorbic acid concentration in nasal lavage DE exposure did not affect antioxidant concentrations in plasma, BW, or BAL DE exposure did not affect malondialdehyde nor protein carbonyl concentrations in plasma or BAL	Oxidative stress and antioxidants
Jiang et al. [21]	PM_2.5_ = 300	None	Asthmatics	DE exposure induced changes in DNA methylation at CpG sites located in genes related to inflammation and oxidative stress, and in miRNA	Systemic inflammation
Xu et al. [22]	PM_1_ = 300	46 dB or 75 dB traffic noise	Healthy	DE exposure associated with symptoms of irritation and decreased peak expiratory flow DE exposure increased inflammatory markers (peripheral blood monocyte and leukocyte counts, serum IL-6)	Systemic inflammation
Channell et al. [23]	PM = 100	None	Healthy	DE or NO_2_ exposure increases circulating proinflammatory factors Plasma from DE or NO_2_ exposed volunteers induced inflammatory response in human endothelial cells	Systemic inflammation
Rabinovitch et al. [24]	PM_2.5_ = 300	None	Asthmatics	DE exposure associated with changes in CysLTR1 methylation and expression CysLTR1 may be involved in mechanistic pathway of DE-related lung function decline in asthmatics	Respiratory
Ryu et al. [25]	PM_2.5_ = 300	Allergen	Atopic + airway hyperresponsive Atopic + airway normally responsive	Short term exposure to allergen + DE alters lung immune regulatory proteins Whole DE associated with decreased allergen-induced levels of SPD in airways Particle depletion restored allergen-induced increase in SPD	Respiratory
Wooding et al. 2020[26]	PM_2.5_ = 300	None	Healthy never-smokers Ex-smokers without COPD Mild-moderate COPD	DE exposure increased neutrophil extracellular traps in lung DE exposure increased peripheral neutrophil activation in COPD patients COPD patients may be more susceptible to inflammation post DE exposure	Respiratory
Mookherjee et al. [27]	PM_2.5_ = 300	Allergen	Atopic + airway hyperresponsive Atopic + airway normally responsive	Co-exposure to DE and allergen associated with protein changes in lung not detected with DE mono-exposure or allergen alone	Respiratory
Clifford et al. [28]	PM_2.5_ = 300	Allergen	Atopic, non-asthmatic Asthmatics	In bronchial epithelium, allergen mono-exposure, DE mono-exposure, or DE + allergen co-exposure induced changes at 7 CpG sites at 48 h post exposure Exposure to allergen and DE separated by 4 weeks associated with changes in over 500 CpG sites Changes modified by which exposure occurred first	Respiratory
Kramer et al. [29]	PM_2.5_ = 300	Allergen	Atopic + airway hyperresponsive Atopic + airway normally responsive	Co-exposure to DE + allergen may cause protective changes in lung adiponectin Protective response not observed after allergen mono-exposure or in participants with baseline airway hyperresponsiveness	Respiratory
Carlsten et al. [30]	PM_2.5_ = 300	Allergen	Atopic + airway hyperresponsive Atopic + airway normally responsive	DE enhanced allergen-induced increases airway eosinophils, IL-5, and eosinophil cationic protein in atopic volunteers GSTT1 null genotype significantly associated with enhanced IL-5 response	Respiratory
Hosseini et al. [31]	PM_2.5_ = 300	Allergen	Atopic + airway hyperresponsive Atopic + airway normally responsive	In atopic volunteers, allergen + DE co-exposure increased CD4, IL-4, CD138, and neutrophil elastase in respiratory submucosa Allergen + DE co-exposure did not change eosinophils or mast cells	Respiratory
Behndig et al. [32]^a^	PM_10_ = 100	None	Healthy Mild asthmatics Moderate asthmatics Allergic rhinitics, non-asthmatic	DE exposure did not affect markers of proliferation and apoptosis in in the bronchial epithelium of asthmatics, allergic rhinitics, or healthy subjects	Respiratory
Larsson et al. [33]	PM_10_ = 100	None	Allergic rhinitics	DE exposure did not induce markers of neutrophilic inflammation in the airways of subjects with allergic rhinitics DE exposure did not increase number of allergic inflammatory cells in airways DE exposure decreased tryptase in the absence of allergic symptoms	Respiratory
Hussain et al. [34]	PM_2.5_ = 300	None	Asthmatics	Acute DE exposure increased airway hyperreactivity and obstruction in asthmatic subjects DE exposure increased nitrite in exhaled breath condensate	Respiratory
Londahl et al. [35]	PM_1_ = 50, 300 (multi-concentration crossover)	None	Healthy COPD	Deposited dose rate of inhaled DEP was higher in subjects with COPD compared to healthy subjects Deposited dose rate increased with increasing disease severity	Respiratory
Behndig et al. [36]	PM_10_ = 100	None	Healthy Mild asthmatics Moderate asthmatics	DE exposure significantly increased neutrophil count, IL-6, and MPO in airways of healthy subjects No neutrophilic inflammation observed in airways of asthmatic subjects	Respiratory
Sehlstedt et al. [37]	PM_10_ = 300	None	Healthy	Exposure to DE increased bronchial adhesion molecule expression and bronchoalveolar eosinophil numbers These effects were found with DE generated from urban running conditions but not with DE from idling conditions	Respiratory
Sawant et al. [38]	PM_2_ = 100	NO_2_	Healthy Asthmatics	Exposure to DE at 100 μg/m^3^ generated in this facility did not cause significant change in lung function tests	Respiratory
Bosson et al. [39]	PM = 300	O_3_	Healthy	DE and O_3_ co-exposure significantly increased sputum MPO and percentage of neutrophils compared to DE mono-exposure MPO response was significantly associated with neutrophils and with MMP-9	Respiratory
Behndig et al. [40]	PM_10_ = 100	None	Healthy	DE exposure increased neutrophil and mast cell numbers in bronchial mucosa DE exposure increased neutrophil numbers, IL-8, and MPO in bronchial lavage These changes were not observed in alveolar lavage	Respiratory
Pourazar et al. [41]	PM_10_ = 300	None	Healthy	DE exposure significantly increased IL-13 in bronchial epithelium DE exposure did not significantly affect IL-10 or IL-18 in bronchial epithelium	Respiratory
Stenfors et al. [42]	PM_10_ = 100	None	Healthy Asthmatics	DE exposure increased airway resistance in both healthy and mild asthmatics DE exposure increased airway neutrophils, leukocytes, and IL-8 in healthy subjects DE exposure did not induce neutrophilic inflammation or exacerbate pre-existing eosinophilic inflammation in airways of asthmatic subjects	Respiratory
Nordenhall et al. [43]	PM_10_ = 300	None	Asthmatics	Acute DE exposure significantly increased airway hyperresponsiveness, airway resistance, and sputum IL-6 in asthmatic subjects DE exposure did not affect sputum methylhistamine, eosinophil cationic protein, MPO, or IL-8	Respiratory
Nightingale et al. [44]	PM_10_ = 200	None	Healthy	Exposure to resuspended DEP did not affect pulse, BP, or lung function DEP exposure increased sputum neutrophils, sputum MPO, and exhaled CO DEP exposure did not affect peripheral blood inflammatory markers	Respiratory
Nordenhall et al. [45]	PM_10_ = 300	None	Healthy	DE exposure significantly increased sputum neutrophils, IL-6, and methylhistamine Percentage of sputum neutrophils was significantly increased at 24 h compared to 6 h regardless of exposure condition	Respiratory
Salvi et al. [46]	PM_10_ = 300	None	Healthy	DE exposure increased IL-8 gene transcription and expression in bronchial tissue DE exposure increased GRO-α expression in bronchial epithelium DE exposure did not significantly affect transcription of IL-1b, TNF-α, IFN-y, or GM-CSF in lung	Respiratory
Salvi et al. [47]	PM_10_ = 300	None	Healthy	DE exposure significantly increased airway neutrophils and B lymphocytes DE exposure increased neutrophils, mast cells, T lymphocytes, ICAM-1, and VCAM-1 in bronchial tissue DE exposure significantly increased peripheral blood neutrophils and platelets	Respiratory
Rudell et al. [48]	n/a	None	Healthy	Lung function not affected by DE exposure DE exposure associated with symptoms such as unpleasant smell, eye irritation, nasal irritation	Respiratory
Tousoulis et al. [49]	PM_2.5_ = 25	None	Healthy non-smokers Healthy smokers	Acute DE exposure associated with adverse effects on endothelial function, vascular walls, and heart rate variability even at 24 h post-exposure DE exposure associated with increased inflammatory markers and abnormal fibrinolytic markers	Cardiovascular
Sack et al. [50]	PM_2.5_ = 200	Antioxidant	Healthy	DE exposure induced acute vasoconstriction in brachial artery Pre-treatment with antioxidant enhanced DE-induced vasoconstriction	Cardiovascular
Langrish et al. [51]^b^	PM_10_ = 300	Carbon nano-particles	Healthy Stable CAD with previous myocardial infarction	Acute controlled exposure to air pollutants (including DE and carbon nanoparticles) did not increase the short-term risk of arrhythmia	Cardiovascular
Tong et al. [52]	PM = 100, 200, 300 (single sequence)	None	Healthy	Acute exposure to DE at 300 μg/m^3^ decreased brachial artery diameter, increased DBP, and induced changes in heart rate variability in GSTM1 null individuals These cardiovascular changes were concentration dependent	Cardiovascular
Krishnan et al. [53]	PM_2.5_ = 200	None	Healthy Metabolic syndrome	Acute DE exposure increased hematocrit and hemoglobin DE exposure increased platelet count in healthy but not metabolic syndrome volunteers Levels of IL-1β, IL-6, MPO, and endothelial activation molecules were increased post-DE exposure	Cardiovascular
Langrish et al. [54]	PM_10_ = 300	NO synthase inhibitor, SNP, ACh	Healthy	DE exposure increased plasma nitrite but this increase was not sufficient to compensate for excess NO consumption BP and central arterial stiffness were increased by systemic NO synthase inhibitor post DE exposure compared to FA	Cardiovascular
Wauters et al. [55]	PM_2.5_ = 300	None	Healthy	Acute DE exposure attenuated vasodilation induced by ACh but not SNP DE exposure increased ROS production in endothelial cells	Cardiovascular
Cosselman et al. [56]	PM_2.5_ = 200	None	Healthy Metabolic syndrome	SBP was increased during and post DE exposure, effect not modified by metabolic syndrome DE exposure did not significantly affect heart rate or DBP	Cardiovascular
Lund et al. [57]	PM = 100	None	Healthy	Acute exposure to DE upregulated atherosclerosis-associated factors such as MMP-9, and ET-1 Effect mediated through oxLDL-LOX-1 receptor signalling DE exposure significantly increased plasma-soluble LOX-1	Cardiovascular
Mills et al. [58]	PM_2_ = 300 PM_2_ = 5 (particle-depleted)	Carbon nano-particles	Healthy	DE exposure increased SBP and attenuated bradykinin/ACh/SNP-induced vasodilation Exposure to pure carbon nanoparticulate or filtered DE did not affect vasodilation DEP but not carbon nanoparticulate attenuated vasorelaxation in vitro	Cardiovascular
Mills et al. [59]	PM = 300	None	Healthy Stable CAD with previous myocardial infarction	Acute DE exposure did not affect heart rhythm or heart rate variability in healthy subjects or subjects stable coronary artery disease	Cardiovascular
Barath et al. [60]	PM_10_ = 250	None	Healthy	DE exposure impaired vasomotor function and endogenous fibrinolysis DE generated from transient running conditions and DE from idling produced similar effects	Cardiovascular
Langrish et al. [61]	PM_10_ = 300	None	Healthy	DE exposure had no effect on plasma ET-1, BP, or heart rate DE exposure increased vascular sensitivity to ET-1 DE exposure attenuated vasodilation induced by ET(A) receptor antagonism	Cardiovascular
Lund et al. [62]	PM = 100	None	Healthy	Acute DE exposure in humans significantly increased plasma ET-1 and MMP-9 expression and activity Gasoline engine exhaust increased circulating and vascular factors associated with atherosclerosis in mice	Cardiovascular
Lundback et al. [63]	PM = 350	None	Healthy	DE exposure associated with immediate and transient increase in arterial stiffness	Cardiovascular
Carlsten et al. [64]	PM_2.5_ = 100, 200 (multi-concentration crossover)	None	Metabolic syndrome	In subjects with metabolic syndrome, DE exposure did not induce significant prothrombotic changes in D-dimer, vWF, and PAI-1	Cardiovascular
Lucking et al. [65]	PM = 350	None	Healthy	DE exposure increased ex vivo thrombus formation and increased in vivo platelet activation	Cardiovascular
Peretz et al. [66]	PM_2.5_ = 100, 200 (multi-concentration crossover)	None	Healthy Metabolic syndrome	Acute DE exposure did not have a consistent effect on autonomic control of the heart	Cardiovascular
Peretz et al. [67]	PM_2.5_ = 100, 200 (multi-concentration crossover)	None	Healthy Metabolic syndrome	Acute DE exposure at 200 μg/m^3^ was associated with vasoconstriction of brachial artery and effect may be dose-dependent Exposure to DE at 200 μg/m^3^ increased plasma ET-1	Cardiovascular
Carlsten et al. [68]	PM_2.5_ = 100, 200 (multi-concentration crossover)	None	Healthy	DE exposure at 100 μg/m^3^ and 200 μg/m^3^ did not induce significant pro-thrombotic changes in D-dimer, vWF, PAI-1, or platelets DE exposure did not significantly affect C-reactive protein	Cardiovascular
Mills et al. [69]	PM_10_ = 300	None	Stable CAD with previous myocardial infarction	In men with previous myocardial infarction, acute DE exposure enhanced ECG changes consistent with myocardial ischemia DE exposure decreased acute release of endothelial tPA	Cardiovascular
Tornqvist et al. [70]	PM = 300	None	Healthy	DE exposure significantly increased plasma TNF-α and IL-6 DE exposure attenuated ACh and bradykinin-induced vasodilation DE exposure had no effect on SNP or verapamil-induced vasodilation	Cardiovascular
Mills et al. [71]	PM = 300	None	Healthy	DE exposure attenuated bradykinin, ACh, and SNP-induced vasodilation DE exposure attenuated bradykinin-induced increase in plasma tPA	Cardiovascular
Cliff et al. [72]	PM_2.5_ = 300	None	Healthy	Acute DE exposure did not affect IL-6, TNF-a, astrocytic protein S100b, neuronal cytoplasmic enzyme neuron-specific enolase, or serum brain-derived neurotrophic factor	Neurological
Cruts et al. [73]	PM = 300	None	Healthy	DE exposure significantly increased median power frequency in the frontal cortex on quantitative EEG DE exposure was associated with general cortical stress response	Neurological
Koch et al. [74]	PM_2.5_ = 300	Inhaled salbutamol	Exercise-induced broncho-constriction	Acute exercise induced microvascular and macrovascular vasodilation Vasodilatory response preserved with DE exposure Heart rate significantly increased after DE exposure compared to FA	Exercise
Giles et al. [75]	PM_2.5_ = 300	None	Healthy	No acute increase in adhesion molecules and inflammatory markers in healthy volunteers during exercise + concomitant DE exposure	Exercise
Giles et al. [76]	PM_2.5_ = 300	None	Healthy	Exercising during DE exposure significantly increased plasma NOx compared to FA ET-1 was significantly decreased at 2 h post-DE exposure compared to FA, effect not modified by exercise intensity No DE-associated changes in FMD or blood pressure	Exercise
Giles et al. [77]	PM_2.5_ = 300	None	Healthy	Exercise associated with increased FeNO, decreased HRV, and increased plasma norepinephrine These exercise-induced changes not modified by DE exposure	Exercise
Wauters et al. [78]	PM_2.5_ = 300	None	Healthy	Exercise during acute DE exposure significantly increased markers of platelet activation (P-selectin and CD63) Acute DE exposure did not impair platelet aggregation during exercise or rest	Exercise
Wauters et al. [79]	PM_2.5_ = 300	Dobutamine stress Exercise in normoxia Exercise in hypoxia	Healthy	DE exposure during high cardiac output increased pulmonary vascular resistance and decreased distensibility of pulmonary resistive vessels	Exercise
Giles et al. [80]	PM_2.5_ = 300	None	Healthy	Respiratory and metabolic responses were greater during low intensity exercise compared to high intensity exercise during DE exposure	Exercise
Giles et al. [81]	PM_2.5_ = 300	None	Healthy	DE exposure significantly decreased exercise-induced bronchodilation DE exposure significantly increased heart rate during exercise DE exposure did not significantly impair performance on 20 km cycling time trial	Exercise
Li et al. [82]	PM_2.5_ = 300	Allergen	Atopic + airway hyperresponsive Atopic + airway normally responsive	Changes in DNA methylation regulation enzymes are involved in response to allergen challenge These changes are dependent on airway hyperresponsiveness, irrespective of DE exposure	Co-exposures
Wooding et al. [83]	PM_2.5_ = 300 PM_2.5_ = 20 (particle-depleted)	Allergen	Atopic + airway hyperresponsive Atopic + airway normally responsive	Co-exposure to DE + allergen associated with impaired lung function Impairment worse with particle depleted, NO_2_ enriched DE	Co-exposures
Biagioni et al. [84]	PM_2.5_ = 300	Allergen	Atopic + airway hyperresponsive Atopic + airway normally responsive	In atopic volunteers, markers of allergic inflammation (SPD and MPO) are increased by allergen exposure but minimally by DE DE decreases levels of protective protein CC16, while allergen has minimal effect	Co-exposures
Rider et al. [85]	PM_2.5_ = 300	Allergen	Atopic	DE or allergen exposure significantly modulate expression of miRNA and genes associated with bronchial immune responses in atopic participants DE did not enhance allergen-associated effects at 48 h	Co-exposures
Zhang et al. 2016 [86]	PM_2.5_ = 300	Allergen	Atopic + airway hyperresponsive Atopic + airway normally responsive	FEV_1_ was significantly decreased post DE and allergen co-exposure in GSTT1 null individuals Post DE and allergen co-exposure, levels of an oxidative stress marker were higher in GSTT1 null individuals compared to GSTT1 present individuals	Co-exposures
Stiegel et al. [87]	PM = 300	O_3_	Healthy	DE and O_3_ co-exposure suppressed plasma IL-5, IL-12p70, IFN-γ, and TNF-α DE and O_3_ co-exposure significantly decreased circulating monocytes and lymphocytes, and significantly increased neutrophils	Co-exposures
Madden et al. [88]	PM = 300	O_3_	Healthy	DE and O_3_ co-exposure decreased FEV_1_ in a greater than additive manner compared to DE mono-exposure and O_3_ mono-exposure	Co-exposures
Barath et al. [89]	PM_10_ = 300	O_3_	Healthy	DE exposure increased FeNO compared to FA O_3_ exposure did not affect FeNO	Co-exposures
Bosson et al. 2008 [90]	PM_10_ = 300	O_3_	Healthy	DE exposure followed by O_3_ exposure increased number of bronchial neutrophils, number of bronchial macrophages, and eosinophil protein X levels	Co-exposures
Hemmingsen et al. [91]	PM_1_ = 300	46 dB or 75 dB traffic noise	Healthy	Exposure to DE was not associated with markers of genotoxicity, oxidative stress or inflammation in PBMC Exposure to traffic noise was associated with markers of DNA damage	Co-exposures
Pawlak et al. [92]	PM = 100	Live attenuated influenza virus	Allergic rhinitics	In volunteers with allergic rhinitis, DE exposure prolongs eosinophil activation induced by influenza virus DE exposure decreased markers of NK cell activation and recruitment	Co-exposures
Noah et al. [93]	PM = 100	Live attenuated influenza virus	Healthy Allergic rhinitics	In allergic rhinitis, acute DE exposure increased eosinophil activation and increased virus quantity post inoculation with influenza virus	Co-exposures
Vieira et al. [94]	PM_2.5_ = 300 PM_2.5_ = 25 (particle-depleted)	None	Healthy Heart failure	Compared to unfiltered DE, particle filtered DE reduced markers of endothelial dysfunction and decreased BNP in subjects with heart failure	Filtered DE
Vieira et al. [95]	PM_2.5_ = 300 PM_2.5_ = 25 (particle-depleted)	None	Healthy Heart failure	Acute DE adversely affected markers of exercise capacity in subjects with heart failure Particle filtered DE mitigated adverse effects of DE exposure on VO_2_ and O_2_ pulse	Filtered DE
Muala et al. [96]	PM_1_ = 350 PM_1_ = 200 (particle- depleted) PM_1_ = 100 (particle- depleted)	None	Healthy	Cabin air inlet particle filter with active charcoal component reduced particulates and gaseous components of DE Cabin filter reduced DE-associated symptoms	Filtered DE
Lucking et al. [97]	PM = 300 PM = 10 (particle- depleted)	None	Healthy	DE exposure reduced vasodilation and increased ex vivo thrombus formation Use of particle trap increased vasodilation, reduced thrombus formation, and increased tPA	Filtered DE
Rudell et al. [98]	PM = 300 PM = 200 (particle- depleted) PM = 100 (particle- depleted) PM = 150 (particle- depleted) PM = 150 (particle- depleted)	None	Healthy, not often exposed to DE Healthy, often exposed to DE	Use of particle filter did not reduce intensity of DE-associated symptoms Use of charcoal filter together with particle filter reduced intensity of symptoms DE-associated symptoms	Filtered DE
Rudell et al. [99]	n/a	None	Healthy	DE exposure increased neutrophils in airway lavage DE induced migration of alveolar macrophages into airways Use of particle trap did not significantly attenuate DE-induced effects	Filtered DE
Rudell et al. [100]	n/a	None	Healthy	DE exposure was associated with irritative symptoms and bronchoconstriction Use of a particle trap did not significantly attenuate these DE-induced effects	Filtered DE
Gouveia-Figueira et al. [101]	PM_10_ = 150	None	Healthy	Biodiesel exhaust exposure was associated with changes in levels of some circulating lipid metabolites, mainly monohydroxy fatty acids	Markers and quantification of DE exposure
Gouveia-Figueira et al. [102]	PM = 150	None	Healthy	Exposure to biodiesel exhaust alters levels of biolipids in BW and BAL samples Exposure to biodiesel exhaust significantly increased levels of PGE_2_, 12,13-DiHOME, and 13-HODE in BAL samples	Markers and quantification of DE exposure
Lu et al. [103]^c^	PM_1_ = 300 PM_2.5_ = 100 (multiple studies)	46 dB or 75 dB traffic noise	Healthy	Acute DE exposure did not significantly alter levels of urinary PAH	Markers and quantification of DE exposure
Wierzbicka et al. [104]	PM_1_ = 300	46 dB or 75 dB traffic noise	Healthy	DE characteristics vary greatly even at the same DEP mass concentration Size dependent effective density prevents overestimation of lung deposited dose Symptoms of nose and eye irritation were present	Markers and quantification of DE exposure
Rissler et al. [105]	PM_10_ = 50, 300 (multi-concentration crossover)	None	Healthy	Deposition of DEP was similar to spherical particles if plotted as a function of mobility diameter Total deposited fraction of DEP is associated with tidal volume and breathing frequency Lung deposition fractions varies greatly between subjects	Markers and quantification of DE exposure
Huyck et al. [106]	PM_10_ = 300	None	Healthy	Urine 1-aminopryine can be used as biomarker of DE exposure There are two subgroups of subjects in terms of timing of 1-aminopryine excretion	Markers and quantification of DE exposure
Hubbard et al. [107]	PM_2.5_ = 100	None	Healthy	Polar VOC in exhaled breath condensates varied with gender and between healthy subjects Most polar VOCs likely of endogenous source	Markers and quantification of DE exposure
Laumbach et al. [108]	PM_10_ = 300	None	Healthy	DE exposure significantly increased urinary 1-aminopryine Large inter-subject variability in urine 1-aminopryine concentration and time-course of detectability	Markers and quantification of DE exposure
Sawyer et al. [109]	PM_2.5_ = 100	None	Healthy	DE exposure did not affect volume or total protein concentration of exhaled breath concentrates	Markers and quantification of DE exposure
Sobus et al. [110]	PM_2.5_ = 100	None	Healthy	Naphthalene and phenanthrene may be useful surrogates for DE concentration	Markers and quantification of DE exposure
Curran et al. [111]	PM_2.5_ = 300	None	Healthy	Non-significant reduction in postural stability after DE exposure	Other
Carlsten et al. [112]	PM_2.5_ = 100, 200 (multi-concentration crossover)	Antioxidant	Healthy Metabolic syndrome	Controlled exposure to DE associated with mild symptoms Majority of participants will not experience any symptoms Blinding to DE exposure is effective	Other
Kipen et al. [113]	PM_2.5_ = 200	Secondary organic aerosol	Healthy	Exposure to DE or secondary organic aerosols induced decline in WBC and RBC proteasome activity	Other
Laumbach et al. [114]	PM_2.5_ = 300	Psychological stressor task	Healthy	DE exposure was associated with small but significant increases in symptom scores Psychological stressor did not increase symptom severity	Other
Pleil et al. [115]	PM_2.5_ = 100	None	Healthy	Heat maps can be used to study existing environmental and biomarker concentrations of PAH	Other

Table is organized by primary topic, then by year (most recent to least recent), then alphabetically by author. Publications categorized under “co-exposure” have been organized by type of co-exposure (eg. allergen, ozone, etc.), then by year and author as above. Target PM concentration used where available—otherwise achieved concentration used instead

*Ach* acetylcholine, *BAL* bronchoalveolar lavage, *BNP* B type natriuretic peptide, *BP* blood pressure, *BW* bronchial wash, *CAD* coronary artery disease, *CAP* concentrated ambient particles, *CC16* club cell secretory protein 16, *CO* carbon monoxide, *COPD* chronic obstructive pulmonary disease, *CysLTR1* cysteinyl leukotriene receptor 1, *DBP* diastolic blood pressure, *DE* diesel exhaust, *DEP* diesel exhaust particles, *12,13-DiHOME* 12,13-dihydroxyoctadecenoic acid, *ECG* electrocardiogram, *EEG* electroencephalogram, *ET-1* endothelin-1, *ET(A)* endothelin receptor A, *FA* filtered air, *FeNO* fraction of exhaled nitric oxide, *FEV*_*1*_ forced expiratory volume in one second, *FMD* flow mediated dilation, *GM-CSF* granulocyte–macrophage colony-stimulating factor, *GRO- α* growth-regulated oncogene alpha, *GSH/GSSG* reduced to oxidized glutathione (ratio), *13-HODE* 13-hydroxyoctadecadienoic acid, *HRV* heart rate variability, *ICAM-1* intercellular adhesion molecule 1, *IFN-γ* interferon-gamma, *IL* interleukin, *LOX-1* lectin-like oxidized low density lipoprotein receptor-1, *miRNA* microRNA, *MMP-9* matrix metalloproteinase-9, *MPO* myeloperoxidase, *NK* natural killer, *NO* nitric oxide, *NO*_*2*_ nitrogen dioxide, *NOx* nitrite and nitrate, *O*_*2*_* pulse* oxygen uptake per heartbeat, *O*_*3*_ ozone, *oxLDL* oxidized low density lipoprotein, *PAH* polycyclic aromatic hydrocarbons, *PAI-1* plasmin activator inhibitor-1, *PGE*_*2*_ prostaglandin E2, *PM* particulate matter, *PM*_*1*_ PM with aerodynamic diameter under 1 μm, *PM*_*2*_ PM with aerodynamic diameter under 2 μm, *PM*_*2.5*_ PM with aerodynamic diameter under 2.5 μm, *PM*_*10*_ PM with aerodynamic diameter under 10 μm, *RBC* red blood cells, *ROS* reactive oxygen species, *SBP* systolic blood pressure, *SNP* sodium nitroprusside, *SPD* surfactant protein D, *TNF-α* tumor necrosis factor alpha, *tPA* tissue plasminogen activator, *VCAM-1* vascular adhesion molecule 1, *VO*_*2*_ maximal oxygen uptake, *VOC* volatile organic compounds, *vWF* von Willebrand factor, *WBC* white blood cells

^a^Behndig et al. [32] is a follow-up study to Behndig et al. [36] and Larsson et al. [33] using archived biopsies

^b^Langrish et al. [51] uses data pooled from multiple publications, including Barath et al. [60], Cruts et al. [73], Mills et al. [59, 69, 71]

^c^Specimens used in Lu et al. [103] were derived from the participants in Pleil et al. [115] (EPA study), Hubbard et al. [107] (EPA study), Sobus et al. [110] (EPA study), Sawyer et al. [109] (EPA study), and Wierzbicka et al. [104] (Lund study).

### Oxidative stress outcomes

#### Diesel exhaust exposure induces oxidative stress

Robust evidence from CHE-DE studies supports oxidative stress as a mechanism of DE-associated effects. DE exposure at 300 μg/m^3^ PM_10_ has been shown to induce activation and nuclear translocation of factors involved in response to oxidative stress, such as nuclear factor (NF)-κB (*p* = 0.02), as well as the mitogen-activated protein kinases p38 (*p* = 0.01) and JNK (*p* = 0.04), in the bronchial epithelium of healthy participants [18]. Other studies have demonstrated changes in gene expression and DNA methylation linked to oxidative stress pathways in peripheral blood mononuclear cells after DE exposure [15, 21].

Studies that demonstrate enhanced effects amongst those with variants of genes of the glutathione-s-transferase (GST) family, and others related to oxidative stress metabolism, further support the role of oxidative stress herein. Some CHE-DE studies estimate genetic susceptibility to oxidative stress by stratifying participants based on GSTM1 status, a gene coding one of several glutathione S-transferases involved in response to oxidative stress [116]. The GSTM1 null phenotype is common and is linked to decreased tolerance to oxidative stress as well as increased lung inflammation [13, 83, 86, 116], though this effect is not always observed [30]. Accordingly, there is evidence from CHE-DE studies that individuals with variant GST-family genotypes are more susceptibile to adverse effects of DE exposure [30], though this area of the literature is not entirely consistent and further investigation is necessary.

#### Potential benefit of supplemental and endogenous antioxidants

The potential of exogenous antioxidants, as supplements, in reducing the harmful effects of DE inhalation has been evaluated in CHE-DE studies as well. As noted above, in participants with baseline airway hyperresponsiveness, exposure to PM_2.5_ at 300 μg/m^3^ (subsequently within this review, this will be abbreviated simply to the mass concentration, e.g. DE300 unless otherwise indicated) increased this hyperresponsiveness, and N-acetylcysteine supplementation for 6 days prior to DE exposure eliminated this effect [13]. Another analysis from this study showed that antioxidant supplementation attenuated DE-induced changes in blood miRNA and associated oxidative stress genes, further implicating such stress as a mechanism for the effects of DE [14]. However, other studies have failed to demonstrate a protective effect of antioxidant supplementation. Another study found that supplementation with N-acetylcysteine and ascorbate prior to DE300 exposure did not abrogate DE-induced changes in markers of oxidative stress [12] and pre-treatment with antioxidant in a separate study was associated with enhancement of DE-induced vasoconstriction [50]. Therefore, while antioxidant supplementation is a relatively convenient and low-cost intervention, its ability to mitigate the adverse effects of DE has has not been consistently validated in CHE-DE studies [117]. It is likely that the uncertainty therein relates to the specific anti-oxidants, their administration (timing and dose), the host phenotype, and the pollution exposure context, amongst other factors.

The ability of endogenous antioxidants to moderate oxidative stress has also been investigated in a CHE-DE study. In healthy participants exposed to DE at 100 μg/m^3^ PM_10_, the endogenous antioxidants ascorbate, urate, and reduced glutathione in the bronchial and nasal airways were not depleted; rather airway reduced glutathione levels were instead elevated (nasal lavage *p* < 0.05, bronchial wash *p* = 0.004) while markers of inflammation were not, suggesting endogenous antioxidant systems in healthy populations may have been mobilized to sufficiently combat DE-associated oxidative stress [19]. Interestingly, a study of participants with metabolic syndrome did not find evidence of oxidative stress or systemic antioxidant responses after exposure to DE200, perhaps as a result of adaptations to chronic oxidative challenge [16]. This heterogeneity prompts further investigation of the effects of anti-oxidants, whether endogenous or exogenously supplied, including potential modification of effect by relevant gene variants. Furthermore, inference from these studies requires careful attention to the overall methods and results for any given investigation.

### Diesel exhaust induces systemic inflammation

CHE-DE studies have reported robust links between acute DE exposure and systemic inflammation. DE exposure at 276 μg/m^3^ PM_1_ was shown to increase peripheral blood leukocyte count (increase of 0.16 ± 1.01 × 10^9^ cells/L from pre-exposure count) and monocyte count (increase of 0.01 ± 0.10 × 10^9^ cells/L from pre-exposure count) compared to FA (*p* = 0.007 and *p* = 0.017 respectively) [22]. The same study also reported a trend towards increased IL-6 after DE exposure (increase of 0.12 ± 0.78 pg/mL compared to pre-exposure, *p* = 0.066 compared to FA) [22]. A 1999 study of healthy volunteers reported significant increases in neutrophil (*p* = 0.04) and platelets (*p* = 0.02) counts in peripheral blood with DE exposure compared to FA, though HLA-DR + lymphocyte count was decreased (*p* = 0.02) [47]. In a 2012 study, plasma obtained from healthy volunteers exposed to DE at 100 μg/m^3^ was incubated with human endothelial cells, and this demonstrated increased endothelial cell expression of the inflammatory marker vascular cell adhesion molecule-1 by 20% (*p* < 0.05) [23]. Notably, one CHE-DE study suggests that COPD patients may be more susceptible to the pro-inflammatory effects of DE exposure [26]. In this study, markers of neutrophil activation such as CD16, CXCR2, as well as percentage of activated neutrophils (+ 12.2% change) in peripheral blood were increased in COPD patients compared to healthy subjects after DE exposure (*p* = 0.006, *p* = 0.002, *p* = 0.046 respectively) [26]. CHE-DE studies have also described DE-induced changes in DNA methylation [21] and gene expression [17] within inflammatory pathways. In sum, CHE-DE studies provide compelling evidence of systemic pro-inflammatory effects from acute DE exposure.

### Few controlled human exposure studies to diesel exhaust examine genotoxicity

The genotoxic effects of DE and its components have been extensively investigated in animal models, in vitro experiments, and observational studies [118–124]. A 2015 CHE-DE study by Hemmingsen and colleagues examined genotoxicity-related outcomes; while there was no statistically appreciable effect of DE exposure on markers of DNA damage, they did report a significant increase (*p* < 0.05) in the level of hOGG1-sensitive sites with exposure to 75 dB of traffic noise, suggesting a genotoxic effect of traffic noise [91]. Currently, there is paucity of CHE-DE studies pertaining to genotoxicity of DE exposure (Table 1). Future CHE-DE studies investigating this topic would greatly strengthen the pre-existing literature, and serve to establish a stronger link between DE exposure and genotoxicity as a mechanism of DE-associated health effects.

### Respiratory outcomes

#### Diesel exhaust increases pulmonary inflammation in healthy subjects

TRAP exposure has been associated with increased asthma exacerbations [125, 126], development of chronic obstructive pulmonary disease (COPD) [127–130], and symptoms of respiratory irritation [131–133]. CHE-DE studies published in the early 2000’s have consistently demonstrated elevations in biomarkers of pulmonary inflammation after DE exposure, providing an explanation for these observations. DE exposure at concentrations of 200 to 300 μg/m^3^ PM_10_ have been shown to acutely increase inflammatory markers such as interleukin (IL)-6, IL-8, IL-13, methylhistamine, neutrophils, and myeloperoxidase (MPO) in the respiratory tract of healthy subjects [41, 44–46]. Interestingly, pulmonary inflammation induced by DE in asthmatics is less clear. A 2011 study found that exposure to DE at 100 μg/m^3^ PM_10_ increased neutrophils (*p* = 0.01), IL-6 (*p* = 0.03), and MPO (*p* = 0.04) in bronchial wash samples from healthy participants while these markers were not elevated in asthmatic subjects [36]. Another study comparing healthy versus asthmatic volunteers reported similar results, with significantly increased airway neutrophils (*p* < 0.05), lymphocytes (*p* < 0.05), and IL-8 (*p* < 0.05) after DE exposure at 100 μg/m^3^ PM_10_ in only the healthy group [42]. While the asthmatic subjects in both studies had significantly elevated levels of eosinophils (*p* < 0.001 [42]; *p* = 0.013 [36]) and mast cells (*p* < 0.05 [42]; *p* < 0.001 [36]) compared to their healthy controls at baseline or after FA exposure, DE exposure did not significantly increase these markers of allergic inflammation in the asthmatic groups [36, 42].

#### Diesel exhaust worsens asthmatic airway function

Though inflammatory markers are not consistently increased by controlled DE in asthmatic subjects, CHE-DE does transiently worsen relevant asthma physiology. Exposure to DE300 has been shown to increase airway hyperreactivity (methacholine PC_20_ = 14.9 mg/mL after DE compared to 19.7 mg/mL after FA, *p* = 0.012) and obstruction (3.3% decrease in FEV_1_% after DE compared to 3.1% increase after FA at 24 h post exposure, *p* = 0.043) in subjects with asthma [34]. Similarly, in a different study of asthmatics, inhalation of DE at 300 μg/m^3^ PM_10_ increased both airway hyperreactivity (methacholine PC_20_ = 1.77 ± 1.35 mg/mL (DE) compared to 3.47 ± 1.36 mg/mL (FA), *p* < 0.001) and resistance (*p* = 0.004) [43]. Increased airway resistance has also been reported with a lower level of DE exposure, at 100 μg/m^3^ PM_10_ (4.1% increase in healthy subjects (*p* < 0.01 DE compared to FA), 6.5% increase in asthmatics (*p* < 0.01 DE compared to FA)) [42]. One proposed mechanism as to how DE exposure impacts the lungs of asthmatics is through oxidative stress. In one study of 16 participants with mild to moderate asthma, nitrite in exhaled breath condensate was increased immediately after DE exposure (*p* = 0.052) [34]. A separate study found that subjects with baseline airway hyperreactivity had a 42% increase in airway responsiveness after DE exposure compared to FA exposure (*p* = 0.03), with this increase abated by prior anti-oxidant supplementation [13]. Interestingly, this same study also showed that anti-oxidant supplementation in individuals with airway hyper-reactivity reduced baseline airway responsiveness by 20% (*p* = 0.001) [13], further implicating the role of oxidative stress. Other mechanistic insight has come through focus on underemphasized pathways [29, 134] and by interrogating the role of epigenetics on lung function [24]. However, the exact mechanisms underlying the impact of DE on asthmatic lungs is still poorly understood and elucidating the differential pulmonary effects of DE is an important focus for further study.

### Cardiovascular outcomes

#### Diesel exhaust exposure impairs vasomotor function

Exposure to air pollution has been consistently associated with cardiovascular morbidity and mortality [3, 135–137]. One pathophysiological mechanism thought to underlie this association is the impairment of vasomotor function. Indeed, multiple CHE-DE studies have demonstrated a link between DE exposure and vascular dysfunction. A study of 30 healthy male participants showed that 1 h exposure to DE, titrated to a nominal concentration of DE300 significantly impaired vasodilation in response to bradykinin (*p* < 0.05), acetylcholine (*p* < 0.05), and sodium nitroprusside (*p* < 0.001) [71], with similar results observed at DE250 in a different study [60]. Two studies that assessed brachial artery diameter reported acute vasoconstriction, a corollary of impaired dilation, after DE200 (decrease in diameter with DE versus FA = 0.11 mm, *p* = 0.01 [67] and 0.09 mm, *p* = 0.03 [50]) [50, 67]. Another study demonstrated impairment of vasodilation at 24 h after DE exposure, implying this effect may persist even into the day following inhalation [70]. Dysfunction of the endothelial nitric oxide (NO) pathway has been implicated in DE-associated vascular dysfunction. In a study with 12 healthy volunteers, DE exposure reduced vasodilation in response to acetylcholine (*p* < 0.01) but not sodium nitroprusside, suggesting DE inhalation only impairs endothelium-dependent vasodilation [55]. The study further demonstrated a correlation between the impairment of vasomotor function and production of reactive oxygen species (ROS), indicating these effects of DE may be mediated by oxidative stress [55]. In another study, DE inhalation increased plasma nitrite concentration (68 ± 48 nmol/L after DE versus 41 ± 32 nmol/L after FA, *p* = 0.006) in healthy volunteers, suggesting increased NO generation as a potential physiological mechanism to the vasoconstrictive effect of DE [54]. The same study also demonstrated a greater increase in blood pressure (*p* = 0.048) and central arterial stiffness (*p* = 0.007) with DE inhalation compared to filtered air (FA) in the presence of systemic NO synthase inhibition, implying that while DE exposure increases NO generation, this is offset by a greater increase in NO consumption leading to an overall reduction in NO bioavailability [54]. The mechanism of reduced NO bioavailability has been further implicated in animal models [138] as well as other CHE-DE studies [61]. While there is strong evidence that DE exerts its vasomotor effects via the NO pathway, the exact mechanism and the potential involvement of other mediators remains uncertain.

#### Diesel exhaust exposure increases likelihood of thrombosis and decreases fibrinolytic capacity

Another mechanism through which DE increases susceptibility to cardiovascular disease is through its effects on fibrinolysis and thrombosis. DE has been shown to suppress bradykinin-induced release of plasma tissue plasminogen activator in healthy volunteers at DE250 and DE300 [60, 71], suggesting DE inhalation impairs endogenous fibrinolytic function. DE exposure has been linked to increased propensity of thrombus formation, as reported in an ex vivo study conducted with blood drawn from healthy volunteers exposed to DE at 350 μg/m^3^ [65]. However, a different study that also involved healthy participants found no difference in thrombotic markers such as D-dimer, von Willebrand factor (vWF), platelets, and plasminogen activator inhibitor-1 (PAI-1) after exposure to DE100 or DE200 [68]. This discrepancy may be due to sample size, as the authors noted their results trended in the expected direction but failed to meet statistical significance [68]. Another reason could be the DE concentration was insufficient to induce a significant prothrombotic effect, as the Lucking study [65] used a concentration of DE at 350 μg/m^3^. Interestingly, in a similar study conducted in volunteers with metabolic syndrome, hypothesized to be more susceptible to cardiovascular risk, again no significant increase in D-dimer, vWF, and PAI-1 was observed after DE100 and DE200 exposure [64]. In these studies from Seattle, it may be that the biomarker approach was insufficiently sensitive, relative to the Badimon chamber technique used routinely in Umea, to thrombotic phenomena. Though there seems to be a general consensus that DE inhalation is prothrombotic, this effect has most observable at DE concentrations at or above 250 μg/m^3^ and further mechanistic detail remains to be elucidated.

#### Diesel exhaust adversely affects heart rate variability and blood pressure

CHE-DE studies have also investigated the effects of DE exposure on measures of cardiac function. A paper by Langrish et al. [51] using data pooled from multiple studies, including a number of CHE-DE studies [58, 60, 69, 71, 73], reported no significant increase in the short term risk of arrhythmia after acute DE exposure in healthy individuals or those with coronary artery disease [51]. CHE-DE studies have also examined other cardiac parameters such as heart rate and blood pressure. A 2014 study found that 2 h exposure to DE at 300 μg/m^3^ increased diastolic blood pressure (DBP) by an average of 5 mmHg (*p* = 0.04), a change that was less pronounced at DE at 100 μg/m^3^ and 200 μg/m^3^ [52]. DE inhalation at 300 μg/m^3^ also decreased indices of the frequency domain of heart rate variability (HRV), a marker of cardiac autonomic function [52]. Of note, the 6 healthy volunteers who participated in this study were null for the GSTM1 gene, a deletion that is associated with increased susceptibility to oxidative stress [52]. Another study involving both healthy and participants with metabolic syndrome found no consistent effect of DE exposure on heart rate variability at DE100 and DE200 [66], perhaps due to the lower concentrations used. A different study found that exposure to DE200 did not significantly affect DBP but did increase systolic blood pressure (SBP) by an average of 4.4 mmHg (95% CI: 1.1, 7.7, *p* = 0.0009) post-exposure [56]. This study, involving both healthy and participants with metabolic syndrome, did not find a significant effect on heart rate [56]. Observational study designs have reported associations between TRAP exposure and both increased blood pressure [139–141] and changes in heart rate variability [142–144]. Though there is a lack of concordance in the specific findings, CHE-DE studies have overall demonstrated an adverse impact of acute DE exposure on heart rate variability and blood pressure.

### Limited evidence from controlled human exposures to diesel exhaust exposure suggest adverse neurological effects

The neurological effects of acute DE exposure have also been explored in a 2008 CHE-DE study. 10 healthy volunteers were exposed to DE at 300 μg/m^3^, with brain activity monitored via electroencephalography (EEG) during and one hour after the exposure [73]. The results of the study demonstrated a significant increase in median power frequency in the frontal cortex during DE exposure compared to FA (*p* < 0.05), with this elevation attributed predominantly to increased fast wave activity (β2) [73]. Elevated β2 is associated with increased cortical stress, and is often seen in patients with neurological and neuropsychological disorders such as post-traumatic stress disorder, traumatic brain injury, and headache [145, 146]. However, the implications of this increased cortical activity on clinical outcomes such as cognition or neurological disease are unclear. Epidemiological studies, animal models, and in vitro experiments have linked air pollution exposure to the development of neurodegenerative diseases such as Alzheimer’s disease [147] and Parkinson’s disease [148, 149]. Long-term exposure to air pollution has been also shown to adversely impact cognitive performance on verbal and math tests [150]. Children are believed to be particularly susceptible to the cognitive consequences of air pollution. Exposure to TRAP has been shown to negatively impact neurobehavioural function in adolescents [151] as well as cognitive development in children [152]. Pathways that have been proposed to mediate the neurological impacts of DE exposure include neuroinflammation and oxidative stress [148, 153], though the exact mechanisms remain unclear. Cruts et al. [73] is the only CHE-DE study to date that has clearly documented concerning neurological impacts of DE exposure; another performed has revealed some preliminarily reassuring results [72, 111] but with major endpoints not yet resulted in detail, precluding complete conclusions. CHE-DE as a paradigm of TRAP exposure is a tool that promises to elucidate not only the clinical effects of DE on neurological function, but also the pathways that mediate these outcomes such that observational data would be given more credence. Given the paucity of research in this area, and the massive public health implications, future CHE-DE studies focus on neurological effects of DE exposure is warranted.

### Exercising during diesel exhaust exposure is generally not harmful in healthy populations

Those living in areas with sub-optimal air quality are often concerned about outdoor exercise due to the risk of breathing in larger quantities of pollutants. The majority of CHE-DE studies have participants alternate between rest and exercise during exposure, in order to simulate real world variations in activity. Exercise is typically done on a stationary bicycle with a modest target ventilation rate. A smaller number of CHE-DE studies have specifically evaluated the effect of exercise during DE exposure on health endpoints. In three papers from the same group, 18 healthy male participants cycled for 30 min at low or high intensity after inhalation of DE300 [76, 77]. While exposure to DE increased plasma NOx (nitrite and nitrate) levels (*p* < 0.05), this effect was not significantly different between different exercise intensities [76]. Exercise during DE exposure did not increase levels of adhesion molecules or systemic inflammatory markers, nor did it affect blood pressure or flow mediated dilation, a measure of endothelial function [75, 76]. Furthermore, the effects of exercise intensity on heart rate variation and norepinephrine were not modulated by DE exposure [77]. A separate study reported no significant difference in parameters of micro- and macrovascular vasodilation after exercise during DE300 exposure compared to FA, suggesting that DE does not impair vascular effects of exercise [74]. Data from observational literature suggests the benefits of exercise typically outweigh the risks of air pollution and that it may not be necessary for most general populations to avoid exercising during periods of increased air pollution except perhaps when extreme [155–159]. However, some epidemiological studies have reported benefits of physical activity are negated in polluted regions, or even an overall adverse effect of exercise in polluted environments [160–163]. Taken together, results from CHE-DE and observational studies imply exercise in areas of air pollution likely imparts beneficial effects in general, however the detrimental impact of air pollution may attenuate the health benefits of activity.

### Co-exposures

The ability of CHE studies to precisely control exposure conditions is a double-edged sword. While this facilitates casual relationships being inferred between focused parameters and health endpoints, the tight elimination of other environmental variables is scarcely encountered in ambient settings. It is common that people are subject to TRAP while going about their daily activities, simultaneously experiencing many other types of exposures, both airborne and not. One way CHE-DE studies have attempted to remedy this issue is by introducing various co-exposures alongside DE. Examples of these co-exposures include aeroallergens, ozone, noise and viruses. These co-exposure studies help delineate interactions between DE with other environmental factors while also enhancing the real-world relevance of CHE-DE exposures, although the full range of potential co-exposure combinations can never be fully captured in a laboratory-based algorithm [154].

#### Diesel exhaust magnifies allergenic effects

The relationship between allergens and TRAP exposure is one of great interest, as both have the potential to exacerbate atopic airway disease and they occur frequently together in many settings. Co-exposures to DE and allergen have been investigated in several more recent CHE-DE studies. In these experiments, participants are first tested for sensitization to common environmental allergens such as house dust mite, grasses, or birch. Participants then undergo a DE or FA exposure, followed by exposure to an allergen to which they demonstrate being already sensitized (either by inhalation or, alternatively, by instillation of allergen or saline into different lung segments via bronchoscopy, a technique known as segmental allergen challenge). Exposure to allergen alone in atopic subjects has been shown to increase airway markers of allergic inflammation, including surfactant protein D (SPD), MPO, and eosinophils, with an additive effect from DE on some but not all of these markers [30, 84]. DE and allergen co-exposure has also been shown to increase non-allergic inflammatory markers such as CD4 (*p* = 0.035), IL-4 (*p* = 0.034), and neutrophil elastase (*p* = 0.031) in submucosal tissue of atopic participants [31]. Inhalation of DE may also inhibit protective responses to allergen-triggered phenomena in the lung. In a 2020 study, atopic participants were exposed to allergen alone, combination of DE and allergen, combination of particle-depleted DE (PDDE) and allergen, and filtered air control [25]. SPD, a protein that modulates pulmonary immune responses, was increased in BAL samples compared to FA-saline control following exposure to allergen only (*p* = 0.02) but not after exposure to combination DE and allergen (*p* = 0.19) [25]. Exposure to the combination of PDDE and allergen restored the protective increase in SPD (*p* = 0.007), suggesting the PM fraction of DE was responsible for suppressed SPD levels [25]. Interestingly, there may be a complex gene-environment interaction that mediates the effects of allergen and DE exposure. A 2016 study found that individuals null for GSTT1, encoding a glutathione S-transferase involved in mitigating oxidative stress, experienced a significantly greater decrease in FEV_1_ after co-exposure to DE and allergen than those with normal GSTT1 (24.5 ± 19.6% decrease (GSTT1 null) compared to 9.2 ± 7.3% decrease (normal *GSTT1*), *p* = 0.001) [86]. CHE-DE studies have also produced evidence that DE and allergen co-exposure often uniquely affects lung gene expression, DNA methylation, and secreted proteins [27, 82, 85], while also showing how some pathways are less effected by the combination [28, 82, 164]. Given that TRAP and aeroallergens are commonly encountered together in urban and suburban environments, delineating the impacts of co-exposure is a relevant and meaningful area of future research. This is particularly important as evidence mounts for greenness as a health-enhancing exposure, as a major caveat therein is the risk for worsening allergenic phenomena when greenness including allergen-rich species in close proximity to TRAP [165]. Species with lower allergenic potential, such as flowering trees [166], are alternatives that can be considered in green space planning, though further investigation is needed to establish if there is indeed a meaningful difference in health outcomes compared with allergen-rich species.

#### Ozone and diesel exhaust have compounding effects

O_3_ is a prevalent component of ambient air pollution and has been shown to negatively impact the cardiovascular and pulmonary systems [167, 168]. Given the near ubiquity of both DE and O_3_ in the air we breathe, several CHE-DE studies have investigated the complex interaction between these two pollutants. DE and O_3_ appear to have compounding effects on airway inflammation. A 2008 paper reported increased bronchial wash neutrophil (5.4 × 10^4^ cells/L (DE and O_3_) versus 3.6 × 10^4^ cells/L (FA and O_3_), *p* = 0.006) and macrophage (8.2 × 10^4^ cells/L (DE and O_3_) versus 7.1 × 10^4^ cells/L (FA and O_3_), *p* = 0.046) numbers in healthy volunteers exposed to DE at 300 μg/m^3^ PM_10_ followed by 0.2 ppm O_3_, compared to FA followed by O_3_ [90]. An earlier paper from this group found increased neutrophils and MPO in sputum samples with combination DE and O_3_ versus combination DE and FA (*p* < 0.05 and < 0.05 respectively) ([39]. A potentially enhanced effect of DE and O_3_ co-exposure on inflammatory cytokines and white blood cell counts was noted by a different study group as well [87]. With respect to lung function, co-exposure to DE and O_3_ has been shown to magnify decreases in FEV_1_ induced by either exposure alone (*p* = 0.057 when comparing change in FEV_1_ post DE and O_3_ co-exposure to sum of FEV_1_ changes post DE mono-exposure and post O_3_ mono-exposure) [88]. Mechanistically, the effects of DE and O_3_ may be mediated through different pathways. Interestingly, exposure to DE at 300 μg/m^3^ PM_10_ but not 0.3 ppm O_3_ has been shown to increase fraction of exhaled nitric oxide (FeNO) (*p* = 0.01 for DE compared to FA), an indicator of airway inflammation [89]. This may be due to FeNO’s reflection of more eosinophilic inflammation (perhaps induced by DE’s complex mixture including organic elements) while ozone-induced inflammation is primarily neutrophilic. Given the potent ability of CHE studies in investigating contributions of co-exposures, this study design has contributed significantly to our understanding of the interplay between DE and O_3_, whilst recognizing that the laboratory setting imperfectly simulates related ambient mixtures.

#### Limited evidence of interactions between diesel exhaust and traffic noise

One CHE-DE study involved co-exposure to traffic noise, with results reported in four of the presently reviewed publications [22, 91, 103, 104]. Transportation-related noise has been associated with cardiovascular disease risk, though the mechanisms are poorly understood [169]. In this CHE-DE study, healthy participants were exposed to DE titrated to 300 μg/m^3^ PM_1_, alongside traffic noise at 48 or 75 dB [91]. Exposure to the higher level of traffic noise, but not DE, was associated with significantly increased signs of DNA damage in peripheral blood mononuclear cells (*p* < 0.05 effect of 75 dB traffic noise) [91]. However, traffic noise did not modulate DE-induced effects on peak expiratory flow and inflammatory markers [22]. As such, evidence on the interaction between DE and traffic noise remains limited.

#### Diesel exhaust may exacerbate allergic inflammation induced by influenza virus

Two CHE-DE studies involved co-exposure to live attenuated influenza virus (LAIV) [92, 93]. Compared to FA, acute exposure to DE at 100 μg/m^3^ prior to intranasal administration of LAIV was shown to increase markers of eosinophil activation (eotaxin-1, *p* = 0.01; eosinophil cationic protein, *p* < 0.01) in subjects with allergic rhinitis, indicating DE may exacerbate LAIV-induced allergic inflammation [93]. A follow up study produced similar results with respect to eosinophil activation and also presented evidence that this effect is mediated by natural killer (NK) cells [92]. DE was shown to significantly decrease IP-10, a marker of NK cell activation (*p* < 0.05 compared to baseline), suggesting DE reduces eosinophil clearance by NK cells [92]. These studies provide novel insight into the interplay between DE, allergic inflammation, and viral infection, demonstrating how increasing complexity integrated into CHE studies can contribute markedly to our understanding of the interaction of TRAP with various environmental factors.

### Use of particle filters may mitigate adverse effects of diesel exhaust

Given the association of fine particulate matter with negative health outcomes, the ability of particulate filters to mitigate the consequences of air pollution has been widely studied. Several CHE-DE papers have found promising data with regards to the beneficial impact of such filters. For brevity, we will use PDDE (particle-depleted DE) to refer to DE which has undergone particle filtration. The FILTER-HF trial investigated the impact of a particle filter on various endpoints in 26 patients with heart failure [94, 95]. Use of the filter reduced DE concentration from DE325 to DE25 but did not affect levels of gaseous DE components [94, 95]. Exposure to unfiltered DE increased levels of B-type natriuretic peptide (BNP) (47.0 pg/mL (FA) versus 66.5 pg/mL (unfiltered DE), *p* = 0.004) and impaired endothelial function (21% decrease in reactive hyperemia index (RHI) during unfiltered DE exposure compared to FA, *p* = 0.002), but these effects were reduced with PDDE (BNP = 66.5 pg/mL (unfiltered DE) versus 44.0 pg/mL (PDDE), *p* = 0.015; 20% increase in RHI during PDDE compared to unfiltered DE, *p* = 0.019) [94]. During a modified version of the 6-min walking test, exposure to unfiltered DE adversely impacted markers of exercise tolerance, such as 6-min walking distance (*p* = 0.03), maximal oxygen uptake (VO_2_) (*p* < 0.001), and oxygen uptake per heartbeat (O_2_ pulse) (*p* < 0.001) [95]. Use of the particle filter was able to reverse some of these DE-induced changes (*p* < 0.001 (VO_2_) and *p* < 0.001 (O_2_ pulse) comparing PDDE to unfiltered DE) [95]. Another study conducted in healthy volunteers showed that filtration through a particle trap was able to reduce the harmful effects of DE inhalation on vasomotor function, thrombus formation, and fibrinolysis (*p* = 0.04, *p* = 0.02, and *p* = 0.03 respectively, for PDDE versus unfiltered DE) [97]. However, evidence from other CHE-DE studies serve as reminders that the particulate portion of DE is not the only mediator of adverse health effects. A 2019 study exposed healthy volunteers to allergen in combination with unfiltered DE or PDDE [83]. The combination of PDDE and allergen impaired lung function to a greater extent than did unfiltered DE with allergen (7.5% greater decrease in FEV_1_ with PDDE and allergen compared to unfiltered DE and allergen, *p* = 0.047) [83]. Notably, the level of NO_2_ in PDDE exposure was greater than that of unfiltered DE exposure (150 ppb compared to 53 ppb, *p* < 0.0001), implying the detrimental effect of particle filtration may have been mediated by NO_2_ enrichment known to occur with some PM-reducing technologies [83].

CHE-DE studies involving activated charcoal filters have attempted to address the additional benefit of controlling the gaseous fraction of DE. In a 2014 study, 30 healthy subjects were exposed to unfiltered DE and DE filtered through two different particle filters, including one filter with an active charcoal component [96]. Levels of PM_10_ were reduced by 47% using the particle filter alone (*p* < 0.001) and by 74% using the filter containing active charcoal (*p* < 0.001) [96]. The combination with charcoal filter, but not the particle filter alone, reduced the levels of NO_2_ by 85% (*p* < 0.001) and of hydrocarbon by 58% (*p* < 0.001) compared to unfiltered DE [96]. Participants reported fewer subjective symptoms after exposure to DE run through the particle filter without charcoal component compared to unfiltered DE; though this was not statistically significant, symptom reduction in the active charcoal filter condition was significant (*p* < 0.05) compared to unfiltered DE) [96]. An earlier CHE-DE study had also reported greater reduction in symptoms with combination particle and charcoal filter compared to particle filter alone [98]. Though particle filters alone seem to have some protective effects, the benefits of filtration may be improved by adding an activated charcoal component.

### No reliable methods of quantifying personal diesel exhaust exposure in controlled human exposure studies

Chambers used in CHE-DE are typically connected to a host of instruments able to precisely measure airborne concentrations of particulates and gaseous co-pollutants. However, it is rather difficult to quantify exactly how much DE is truly inhaled as this depends on a multitude of participant-dependent factors such as variations in respiratory rate and tidal volume (even when design attempts to control and keep these consistent, due to individual anatomy and other factors). Development of methods to most accurately quantify personal exposure to DE are ongoing, but several CHE-DE studies have assessed the utility of specific biomarkers as surrogate indicators of DE exposure, while recognizing that variability in individual factors related to metabolism makes such markers imperfect proxies for proximal exposure. In a preliminary study, the airborne concentrations of DE constituents that can be found in biological fluids were measured during exposure to DE100 [110]. Naphthalene and phenanthrene, two polycyclic aromatic hydrocarbons (PAHs), were identified as potential markers of DE exposure and were further investigated in a follow up study. The urinary concentrations of these two compounds along with 12 other PAHs were measured prior and after DE exposure ranging from DE106–DE276, but none of these compounds were found to be a suitable biomarker [103]. Blood concentrations of plasma PAHs from the Sobus et al. 2008 [110] study were visualized using a heat map in a separate paper [115], demonstrating a novel approach to displaying data generated in CHE-DE studies. Another compound that has been assessed for this purpose is 1-nitropyrene, a marker of DE exposure that is excreted in urine as 1-aminopyrene [108]. Urinary concentration of 1-aminopyrene in healthy volunteers exposed to DE at 300 μg/m^3^ PM_10_ increased upon DE exposure [108]. However, the utility of this marker is limited by a high degree of variability between subjects [108], likely related to the aforementioned constraint (given inevitable co-exposures to other products of common combustion). While a consistent marker of DE exposure has yet to be found, exploration of this area should continue as identification of such a compound would greatly benefit air pollution research.

### Controlled human studies to diesel exhaust: limitations and future directions

Though CHE-DE studies are important tools in research aiming to assess health effects of TRAP, these experiments carry an inherent set of limitations. As discussed earlier, CHE studies are, by necessity, constrained environments and thus unable to fully replicate the complex co-exposures encountered in the real world. This is remedied to some extent by the increasing sophistication of co-exposures in CHE-DE studies, but the addition of each additional exposure layer imposes further budgetary costs, participant burden, and analytical complexity. CHE studies using ambient pollution [170–172] as well as concentrated ambient particles [173–177] have also been employed to better reflect the intricacies of real world exposures. Another significant weakness of CHE-DE studies is the inherent uncertainty in terms of how the setting relates to the long-term effects of DE exposure. While chronic effects are inevitably the result of a series of acute exposures, the precise relationship between the short and long terms therein is a complex subject of ongoing investigation. Observational studies have provided robust evidence for the detrimental effects of chronic diesel exhaust exposure across multiple physiological systems [178]. One important avenue therein is to integrate or compare the results of CHE studies with data obtained via observational methods, and examples of this approach are emerging [179], albeit with methodological challenges that need further refinement.

Another noteworthy limitation is that the concentrations of DE used in CHE-DE studies are typically on the high end relative to real world exposures. This has typically been motivated by a desire to quantify significant changes in endpoints within a relatively short amount of time, by a need to induce an effect detectable beyond the background exposure levels that participants will routinely experience in daily life, and because a focus on mechanistic insight typically justifies some excursion from typical levels. Furthermore, it is logistically impractical and costly for CHE studies to bring participants in for multiple low-level exposures over an extended period. While the cohort and longitudinal designs often used in studies of chronic TRAP exposure come with their own set of advantages and disadvantages, the approach of collating insight from various study designs—each with their valuable angles of insight—remains the gold standard for ultimate decision-making.

Studies designed to allow assessment of concentration–response relationships can be informative as the whether or not concentrations typical of CHE are exceptional [180]. For example, one study exposed healthy participants to whole DE at 100 μg/m^3^, 200 μg/m^3^, 300 μg/m^3^ and found exposure at a concentration of 300 μg/m^3^, but not the lower concentrations induced cardiovascular responses [52]. While this study might be interpreted as 300 μg/m^3^ being a minimal concentration to induce measureable effects in this specific context, the concentration–response function likely varies according to participant phenotype, details of exposure protocol, and particular outcomes. Indeed, another study examined the effects of 2-h exposures of DE at a modest concentration of DE25 and demonstrated significant differences in measurements of endothelial function and fibrinolysis [49]. And yet, in contrast, Giles et al. [64] used the same measure of endothelial function as Tousoulis et al. [118] but did not find effect of DE exposure, despite using a concentration of DE300 and a shorter exposure duration of 30 min [76]. In epidemiological studies as well, the minimal concentration required to demonstrate discernible effects of chronic PM exposure is unclear. One large scale multicenter European study reported an increase in natural cause mortality in participants chronically exposed to PM_2.5_ concentrations under 20 μg/m^3^ [181]. Furthermore, even at the same chamber concentration of DE, inter-individual variability in the quantity of DE inhaled likely plays a role clouding the threshold concentration for observable effects. As discussed above, a reliable marker of DE exposure has yet to be identified and research in this area is still ongoing. A method of accurately quantifying personal DE exposure would, among its many other potential applications, greatly aid in elucidating the minimum concentrations needed for detection of various endpoints in both CHE-DE and observational studies. Thus, the minimum concentration needed to produce observable or significant effects likely depends not only on the health outcome being investigated, but factors related to study design and participants as well.

As noted above, there is also a lack of consistency among the findings of CHE-DE studies investigating heart rate and blood pressure, though in general CHE-DE studies have revealed a negative effect of acute DE exposure on cardiovascular function. Observational study designs have demonstrated associations between TRAP exposure and both increased blood pressure [139–141] and changes in heart rate variability [142–144]. A 2015 study utilizing CHE to ambient air pollution reported changes in heart rate variability as well [172]. Inter-study variability in the results of CHE-DE studies may indicate that exposures within such protocols are not sufficiently long and/or reflective of complex real-world ambient aerosols to produce a significant and detectable effects, or simply that effects of acute TRAP exposures are distinct from those observed in epidemiology over a longer time course.

Out of an abundance of caution in CHE-DE studies, those with significant medical comorbidities have often been excluded from participating, particularly in the earlier era of such studies. Indeed, results from the small number of CHE-DE studies involving subjects with notable medical conditions have suggested these individuals may respond differently to TRAP exposure relative to healthy populations. As noted previously, in a recent study, patients with mild to moderate COPD were shown to have more activated peripheral neutrophils after acute DE300 exposure compared to healthy never-smokers, suggesting this cohort is more susceptible to the inflammatory effects of DE inhalation [26]. Another CHE-DE study of COPD patients proposed differential deposition of DE particles in the respiratory tract as a cause of their apparent risk in the setting of TRAP [35]. The rate of deposited DE particles during spontaneous breathing was higher in the COPD group versus healthy controls and was also correlated with increasing disease severity [35]. Participants with significant cardiovascular disease have been included in a few studies, such as the FILTER-HF trial reviewed earlier [94, 95]. Another study exposed 20 males with prior myocardial infarction (MI) and stable coronary artery disease to DE at 300 μg/m^3^ PM_10_ [69]. DE inhalation decreased bradykinin-induced release of tissue plasminogen activator in these participants with cardiovascular disease [69]. However, vasodilation in response to acetylcholine was impaired in the MI group compared to healthy controls [69]. Taken together, these results suggest DE exposure in men with stable coronary artery disease may exacerbate myocardial ischemia and impair fibrinolysis [69].

Notably, while this limited set of studies confirms some effects of DE inhalation in these groups, consistent with their chronic cardio-pulmonary disease, these effects have been sub-clinical, anticipated by the study design, and recognized as tolerable by approving ethics boards. Furthermore, and most importantly, they were unassociated with adverse clinical events, across more than a thousand of participants of various phenotypes, and are instead broadly recognized as important advances in our understanding of the pathophysiology of these diseases in the context of a nearly ubiquitous exposure of global concern. Moreover, from a public health standpoint, illustrating the biological plausibility of previously observed consequences of pollution has supported and buttressed the establishment of stronger air quality regulations.

While distinct adverse clinical events from CHE-DE have not reported, studies have noted that undesirable symptoms were more frequent with DE exposure at a concentration of 300 μg/m^3^ PM_1_ [22] and at DE at 300 μg/m^3^ PM_2.5_ [114] compared to filtered air. However, another study, notable for having demonstrated effective blinding to exposure (importantly, as nominal blinding is much more common), suggested that symptoms may be more related to the perception of exposure rather than the actual exposure itself [182], an effect that is especially relevant for self-reported measures. Future studies should not only be designed in a rigourously blinded manner but also assess specifically for effectiveness of that blinding.

Finally, all systems for CHE-DE studies to date have been within a contained infrastructure that is not exposed to the typical ambient environment and associated photochemistry known to ‘age’ the aerosol, in a manner that generally increases oxidative potential. While the use of ambient air pollution in CHE studies does account for this aging effect to an extent, studies that incorporate a method of photochemical aging mimicking that of common urban airsheds would provide yet another incremental step towards optimizing the translational capacity of these already highly informative investigations.

## Conclusion

CHE-DE studies have contributed greatly to our current understanding of health outcomes linked to TRAP exposure, particular in terms of elucidating mechanisms that substantiate—or in some cases put into question—observations from other study contexts. Research has focused on the cardiovascular and pulmonary impacts of DE inhalation, with oxidative stress thought to be the dominant mechanism of DE-induced effects, while other systems such as neuro-cognitive have been more recently explored. Co-exposure studies have delineated powerful interactions between DE and commonly encountered environmental factors, such as allergens and ozone. Particle filters, particularly in combination with activated charcoal filters, are a promising method of reducing the detrimental impacts of TRAP. To date, no robust biomarker of DE exposure has been identified. The main limitation of CHE-DE studies is their inability to directly examine chronic effects of DE inhalation, a niche better filled by other experimental designs [183–186]. While few CHE-DE experiments have included participants with significant medical co-morbidities, evidence from those that have suggests this can be done safely and can reveal aspects of pathophysiology particular to these populations. CHE-DE studies have proven to be an invaluable research tool and continue to advance relevant and applicable knowledge as we strive to further limit exposure to, and adverse effects of, air pollution.

## Data Availability

Not applicable.

## Abbreviations

BNP: B-type natriuretic peptide
CHE: Controlled human exposure
CHE-DE: Controlled human exposure to diesel exhaust
CO: Carbon monoxide
COPD: Chronic obstructive pulmonary disease
DBP: Diastolic blood pressure
DE: Diesel exhaust
EEG: Electroencephalography
FA: Filtered air
FeNO: Fraction of exhaled nitric oxide
HRV: Heart rate variability
IL: Interleukin
MI: Myocardial infarction
MPO: Myeloperoxidase
NF: Nuclear factor
NK: Natural killer cells
NO: Nitric oxide
NO_2_: Nitrogen dioxide
NO_x_: Nitrite and nitrate
O_3_: Ozone
PAH: Polycyclic aromatic hydrocarbons
PAI-1: Plasminogen activator inhibitor-1
PDDE: Particle depleted diesel exhaust
PM: Particulate matter
PM_1_: PM with aerodynamic diameter under 1 μm
PM_2.5_: PM with aerodynamic diameter under 2.5 μm
PM_10_: PM with aerodynamic diameter under 10 μm
RHI: Reactive hyperemia index
SBP: Systolic blood pressure
SPD: Surfactant protein D
TRAP: Traffic related air pollution
VO_2_: Maximal oxygen uptake
vWF: Von Willebrand factor
WHO: World Health Organization
